# Oral Intake of Enzymatically Decomposed AP Collagen Peptides Improves Skin Moisture and Ceramide and Natural Moisturizing Factor Contents in the Stratum Corneum

**DOI:** 10.3390/nu13124372

**Published:** 2021-12-06

**Authors:** Kyoungmi Jung, Seung-Hun Kim, Kyung-Mi Joo, Sung-Hwan Lim, Jin-Hee Shin, Jonghwa Roh, Eunjoo Kim, Chan Woong Park, Wangi Kim

**Affiliations:** 1 Amorepacific Research and Development Center, 1920, Yonggu-daero, Giheung-gu, Yongin 17074, Korea; shkim1228@amorepacific.com (S.-H.K.); kmjoo@amorepacific.com (K.-M.J.); limsungh@amorepacific.com (S.-H.L.); rohjh@amorepacific.com (J.R.); eon827@amorepacific.com (E.K.); woongpc@amorepacific.com (C.W.P.); katemina@amorepacific.com (W.K.); 2 P&K Skin Research Center, 25, Gukheo-daero 62-gil, Yeongdeungpo-gu, Seoul 07236, Korea; jh.s@pnkskin.com

**Keywords:** collagen peptide, skin hydration, skin texture, ceramide, natural moisturizing factor

## Abstract

The stratum corneum (SC) is the outermost layer of the epidermis and plays an important role in maintaining skin moisture and protecting the skin from the external environment. Ceramide and natural moisturizing factor (NMF) are the major SC components that maintain skin moisture. In this study, we investigated whether the oral intake of enzymatically decomposed AP collagen peptides (APCPs) can improve skin moisture and barrier function by assessing changes in the ceramide and NMF contents in the SC after APCP ingestion with the aim to develop a skin functional food. Fifty participants orally ingested APCP (1000 mg) or placebo for 12 weeks, and then, skin hydration and skin texture were evaluated. SC samples were collected to analyze skin scaling, ceramide, and NMF contents. Participants in the APCP group exhibited improved skin moisture content by 7.33% (*p* = 0.031) and roughness by −4.09% (*p* = 0.036) when compared with those in the placebo group. NMF content; the amounts of amino acids (AA), including glycine and proline; and AA derivatives were significantly increased in the APCP group (31.98 μg/mg protein) compared to those in the placebo group (−16.01 μg/mg protein) (*p* = 0.006). The amounts of total ceramides and ceramide subclasses were significantly higher in the APCP group than in the placebo group (*p* = 0.014). In conclusion, our results demonstrate that APCP intake improves skin moisture and increase the ceramide and NMF contents in the SC, thereby enhancing the skin barrier function.

## 1. Introduction

The skin acts as a primary barrier protecting the body from the external environment and preventing the water loss from the body. In particular, the stratum corneum (SC; the uppermost layer of the skin) is crucial for the skin barrier function. The SC consists of multiple layers of flattened corneocytes surrounded by a cornified envelope and embedded in an intercellular lipid matrix. The structure of the SC is generally described by the “brick and mortar” model, in which corneocytes and intercellular lipids represent the bricks and mortar, respectively [1,2]. The SC contains a complex mixture of low-molecular-weight, water-soluble compounds termed natural moisturizing factors (NMFs). NMFs are produced through the proteolysis of filaggrin and desmosomal degradation. NMF consists of hydrophilic amino acids (AAs) and their derivatives (AADs), such as pyrrolidone carboxylic acid (PCA) and urocanic acid (UCA), lactic acid, sugars, organic acids, peptides, and urea, which contribute to the establishment of a water gradient in the SC [3,4,5]. The intercellular lipids present in the SC play an important role in the regulation of the water content. The major skin lipids are ceramides, cholesterol, and free fatty acids (FAs) [6]. The most important lipids that form this permeability barrier are ceramides [7]. Reductions in the NMF and ceramide contents lead to dry skin due to an increase in transepidermal water loss (TEWL) and a decrease in the flexibility of the SC [8,9]. Dry skin accelerates skin aging due to weakening of the skin barrier and aggravates skin diseases, such as atopic dermatitis (AD), xerotic eczema, and winter xerosis [10,11,12,13]. Dry skin becomes more evident with advancing age and is accompanied by decreases in the NMF and ceramide contents [8,14]. Several studies have shown that the application of products containing humectants, such as urea, glycerol, and physiological lipids of the skin, is helpful in protecting skin that is deficient in ceramides and NMF [15,16,17]. This finding implies that increasing the NMF and ceramide contents helps moisturize the skin. Recently, skin moisture improvement through a combination of dietary supplements, and topical humectants have been actively researched. Collagen is a major component of skin-moisturizing dietary supplements. Collagen peptides have been widely used in cosmetics to prevent skin aging and to maintain the integrity of the skin [18]. Recently, collagen peptides have been studied as dietary supplements [19,20,21].

Collagen is a major structural protein of the extracellular matrix. It provides tensile strength and maintains cellular structure [22]. Skin aging induces structural changes in the extracellular matrix, such as decreases in the amount and length of collagen fibers. This reduces the elasticity of the dermal layer and causes deep wrinkles and dryness of the epidermal layer [23]. As skin dryness is directly related to internal or external aging, various agents are being developed to increase skin moisture, and collagen is one of their main ingredients [24]. When enzymatically hydrolyzed collagen peptides are ingested, they circulate in the blood in the form of dipeptides, such as glycine-proline (Gly-Pro) and proline-hydroxyproline (Pro-Hyp), or tripeptides, such as Gly-Pro-Hyp [25,26,27]. Collagen-derived peptides are distributed to the tissues via the bloodstream and accumulate in the skin in the form of peptides or AAs and exert chemotactic activity on the skin fibroblasts [28,29]. When dipeptides containing Hyp, such as Pro-Hyp, are absorbed into the skin, they promote the growth of dermal fibroblasts and increase the production of hyaluronic acid, thus increasing the moisture content in the SC [26,30]. In addition, collagen peptides promote collagen synthesis at the mRNA and protein levels [31,32] and induce the production of strong collagen fibrils to reinforce the skin barrier [33]. Several studies have suggested that the main etiological factor of dry skin conditions, such as AD, is the reduction of ceramide content [11,34]. Ceramide content was decreased in mouse model of skin photoaging, in which skin barrier damage was induced and remained similar to the normal group when fish collagen was administered [35]. Treatment of three-dimensional cultured human skin models with collagen peptides increased the transcript-level expression of ceramide synthases, such as serine palmitoyltransferase-2 and β-glucocerebrosidase [36]. These results suggest that collagen retained the total ceramide content by maintaining lipid profile in the skin [35]. In addition, collagen peptide intake restored hyaluronic acid synthase and skin moisture levels reduced by ultraviolet irradiation and induced filaggrin production, which is pivotal for skin barrier recuperation [37]. Filaggrin increases the AA and AAD contents, which are main components of NMF in the SC [38,39,40].

Based on the above findings, the ingestion of collagen peptides is expected to increase skin moisture by inducing ceramide synthesis and increasing NMF levels in the SC. Therefore, this study aims to confirm the effect of changes in ceramide and NMF contents in SC, owing to the ingestion of enzymatically decomposed AP collagen peptides (APCPs), on skin moisture and skin barrier improvement.

## 2. Materials and Methods

### 2.1. Supplement Preparation

APCPs were provided by Aestura Corp. (Ansung, Korea) and were manufactured by the enzymatic degradation of gelatin derived from the scales of *Nemipterus virgatus*. The APCPs contained >15% tripeptides and 3% Gly-Pro-Hyp. All the supplements were produced following Good Manufacturing Practice at Hazard Analysis Critical Control Points-certified plants. A thousand milligrams of APCP was suspended in purified water with vehicle materials in a 25-mL ampoule at 60 °C for 30 min and sterilized by exposure to ultra-high temperature. The vehicle materials were fruit concentrate, citric acid, trisodium citrate, flavor, sweeteners, gum, stabilizer, and vitamin C. The APCP supplements and placebo were prepared with the same flavor and taste.

### 2.2. Study Design and Ethics

This double-blind, randomized, parallel, and placebo-controlled study was conducted at the P&K Skin Research Center (Seoul, Korea) between December 2020 and March 2021, according to the principles of good clinical practice. The study protocol was approved by the Institutional Review Board of the P&K Skin Research Center (P2012-1602) and was fully explained to all participants, who gave their written informed consent before participation. The study protocol was registered with ClinicalTrials.gov (NCT05059197).

### 2.3. Study Participants

The study participants were men and women (age range, 35–60 years) with dry skin, a skin moisture content of 49% or less, or crow’s feet greater than grade 3 [41]. The exclusion criteria were as follows: (1) presence of skin diseases, such as AD and psoriasis; (2) treatment with any external application or oral dosing containing steroids for a skin disease for longer than 3 months; (3) presence of skin abnormality in the test area, including moles, acne, erythema, dilated capillaries, or sensitive or hypersensitive skin; (4) administration of any dietary supplements, functional foods, or medicines, which could have the same or similar effects as the collagens evaluated in this study; (5) possible development of an allergic reaction to food; (6) acute or chronic physical diseases, including non-controlled metabolic disease; (7) pregnancy or breast-feeding, or a planned pregnancy during the study period; (8) participation or intended participation in any other clinical trial; and (9) deemed unsuitable for participation by the investigator. In total, 50 participants who met all inclusion criteria were included in the study.

### 2.4. Study Procedure

Equal numbers of participants were randomly assigned to APCP and placebo groups. Group allocations were coded and concealed from the participants, laboratory technicians, and study investigators throughout the study. The randomized code was unblinded once all primary statistical analyses were completed. All participants consumed the assigned product once daily for 12 weeks. Efficacy endpoints were assessed at baseline and 12 weeks after the initiation of supplementation. The same skin area was examined and by the same methods at each visit. The skin condition was evaluated after washing and stabilization of the skin in a room with controlled temperature (22 ± 2 °C) and humidity (50% ± 10%) for 30 min. For safety evaluation, laboratory tests, vital sign evaluation, pregnancy testing, and physical measurements were performed at baseline and 12 weeks after the initiation of supplementation, and adverse events were assessed during the intervention period.

### 2.5. Evaluation of Skin Hydration and Transepidermal Water Loss

Skin moisture was measured locally on the anterior forearm 10 cm distal to the wrist using a MoistureMeter D Compact (Delfin Technologies, Ltd., Kuopio, Finland), which measures the skin tissue dielectric constant noninvasively and converts it within seconds into a % skin water content on a scale from 0% to 100%. An increase in the measured value indicates an improvement in skin moisture. TEWL from the forearm was measured using a vapometer (SWL4001, Delfin Technologies Ltd., Kuopio, Finland), which harbors a humidity sensor in a cylindrical measurement chamber that records changes in relative humidity inside the chamber during the measurement and automatically calculates TEWL (g/m^2^h). A decrease in the measured value indicates an improvement in TEWL. All skin moisture and TEWL measurements were performed three times at each visit, and the average values were used for skin hydration analysis.

### 2.6. Evaluation of Skin Texture

To evaluate the skin texture in function of the increase in skin moisture, we measured skin roughness and skin gloss on the cheek at the point where a vertical line from the pupil and a horizontal line from the base of the nose met. Skin roughness was measured using the PRIMOS CR Small Field (GFMesstechnik GmbH, Berlin, Germany), and the average roughness (Ra) was analyzed using the PRIMOS software (version 5.8E). A decrease in the measured Ra value indicates an improvement in skin roughness. Skin gloss was measured on the cheek using a skin glossmeter (Delfin Technologies Ltd., Kuopio, Finland). Skin gloss was measured three times, and the average values were used for analysis. An increase in the measured value indicates an improvement in skin gloss.

### 2.7. Evaluation of SC Flexibility

To evaluate the flexibility of the SC, SC samples were collected from the forearm by tape stripping using D-squame^®^ tape (Cuderm Corp., Dallas, TX, USA) with a diameter of 22 mm. Tape stripping was repeated 11 times. The first sample was imaged using a Visioscan VC98 instrument (Courage+Khazaka Electronic GmbH, Cologne, Germany), and the desquamation index was calculated from the measured values.

### 2.8. Protein Analysis Using the Tape Strips

The remaining 10 stripped tapes were cut in two equal parts, and proteins were extracted from one half. The tapes were placed in glass vials containing 1 mL of 0.1% (*w*/*v*) sodium dodecyl sulfate and 2% (*w*/*v*) propylene glycol in phosphate-buffered solution and sonicated for 1 h to obtain the soluble proteins. The solutions were centrifuged at 15,200× *g*, 4 °C for 5 min. Proteins in the supernatant were quantified using a BCA protein assay kit (Pierce, Rockford, IL, USA). The samples were incubated at 37 °C for 30 min, and then, the absorbance at 562 nm was measured using a microplate reader (Spectramax190; Bio-Rad Laboratories, Hercules, CA, USA). After the measurement, the solutions were stored at 4 °C for later quantification of the AAs and AADs.

### 2.9. Analysis of AAs in the SC

Three AAs, namely l-Asparagine, l-glutamine, and l-tryptophan (Sigma-Aldrich, St. Louis, MO, USA), were dissolved in 0.1 M HCl at a concentration of 200 μg/mL, mixed with standard solutions of seventeen AAs (l-alanine, l-arginine, l-aspartic acid, l-cystine, l-glycine, l-histidine, l-isoleucine, l-leucine, l-lysine hydrochloride, l-methionine, l-phenylalanine, l-proline, l-serine, l-threonine, l-tyrosine, l-valine) (1 nmol/μL in 0.1 M HCl, Agilent Technologies, Santa Clara, CA, USA), and serially diluted to obtain standard solutions of five concentrations for preparing the calibration curves. AA analysis was conducted using the AccQ·Tag derivatization method. Quantitative analysis was performed using an ACQUITY UPLC system (Waters Corp., Milford, MA, USA) equipped with an AccQ·Tag Ultra C18 column (2.1 × 100 mm, 1.7 μm) for chromatographic separation. The mobile phase was 10% AccQ·Tag Ultra concentrate solvent A, whereas eluent B was 100% AccQ·Tag Ultra solvent B. The elution gradient was as follows: 0–0.54 min (99.9% A), 3.50 min (98.0% A), 5.74 min (90.9% A), 7.74 min (78.8% A), 8.20–8.50 min (40.0% A), and 8.60–10.5 min (99.9% A). The flow rate was 0.7 mL/min, and the injection volume was 1 μL. The photodiode array detector was set at 260 nm. Data were processed using the Waters Empower Pro software.

### 2.10. Analysis of AADs in the SC

Four AADs, namely 2-pyrrolidone-5-carboxylic acid (PCA), *trans*-UCA, *cis*-UCA, and citrulline (Sigma-Aldrich, St. Louis, MO, USA), were quantified according to a previously reported UPLC-MS/MS method [42]. Briefly, the sample solution was analyzed using an ACQUITY UPLC-Xevo TQ-S triple quadrupole mass spectrometer (Waters Corp., Milford, MA, USA) equipped with an ESI source. The mass spectrometer was operated in the positive ESI mode under the following operating conditions: capillary voltage, 3.5 kV; ion source temperature, 120 °C; desolvation temperature, 350 °C; desolvation gas flow rate, 750 L/h; and cone gas flow rate, 150 L/h. For data acquisition and processing, MassLynx Version 4.1 (Waters Corp., Milford, MA, USA) was used. NMF content was evaluated as the sum of the contents of AAs and AADs.

### 2.11. Lipid Extraction and Analysis of the Ceramide Content in the SC

The second half of the tape was used for lipid extraction. The tapes were immersed in 3 mL of methanol/ethyl acetate (2:1, *v*/*v*) and sonicated for 10 min. The organic layer was transferred into another tube and evaporated to dryness using a SpeedVac (EZ-2 Plus; Genevac, Switzerland) at 40 °C. The residues were dissolved in 200 µL of methanol/chloroform (2:1, *v*/*v*) and vortexed for 5 min, followed by centrifugation at 17,800× *g*, 4 °C for 5 min for UPLC-MS/MS analysis (ACQUITY UPLC-Xevo TQ-S, Waters Corp.). Four subclasses of ceramides, namely a non-hydroxy FA conjugated to phytosphingosine (NP), a non-hydroxy FA conjugated to sphingosine (NS), a non-hydroxy FA conjugated to dihydrosphingosine base (NDS), and α-hydroxy FA conjugated to phytosphingosine (AP; Avanti Polar Lipids, Alabaster, AL, USA), were quantified according to a previously described UPLC-MS/MS method [43]. Total ceramide content was evaluated as the sum of the contents of four subclasses of ceramide.

### 2.12. Statistical Analysis

The sample size was calculated using the G*Power software (version 3.1.9.6) based on the changes in skin moisture in our previous studies. With an estimated 80% power and 5% significance, the minimum sample size required was 20 participants in each group. Statistical analyses were performed using the SPSS Statistics software (version 19.0; IBM, Inc., Armonk, NY, USA) and JMP^®^ ver15.1.0 (SAS Institute, Inc., Cary, NC, USA). The data were tested for normality using the Shapiro–Wilk test. The baseline measurements were assessed using the independent *t*-test or Mann–Whitney *U* test, according to normality. The difference in the numbers of men and women between groups was analyzed using a chi-square test. Differences from baseline within the group were analyzed using a paired *t*-test or Wilcoxon signed-rank test for nonparametric data. The relative changes in skin parameters were statistically evaluated using an independent *t*-test or Mann–Whitney *U* test. Changes in the amounts of AAs, AADs, total NMF, and ceramides were statistically evaluated using the independent *t*-test or Mann–Whitney *U* test. To analyze the total ceramide improvement rate, differences between the APCP and placebo groups were analyzed using a chi-square test. Pearson’s correlation analysis was performed to determine the correlation between the changes in ceramide and TEWL. *p* < 0.05 was considered significant.

## 3. Results

### 3.1. Study Participants

**Figure 1:**
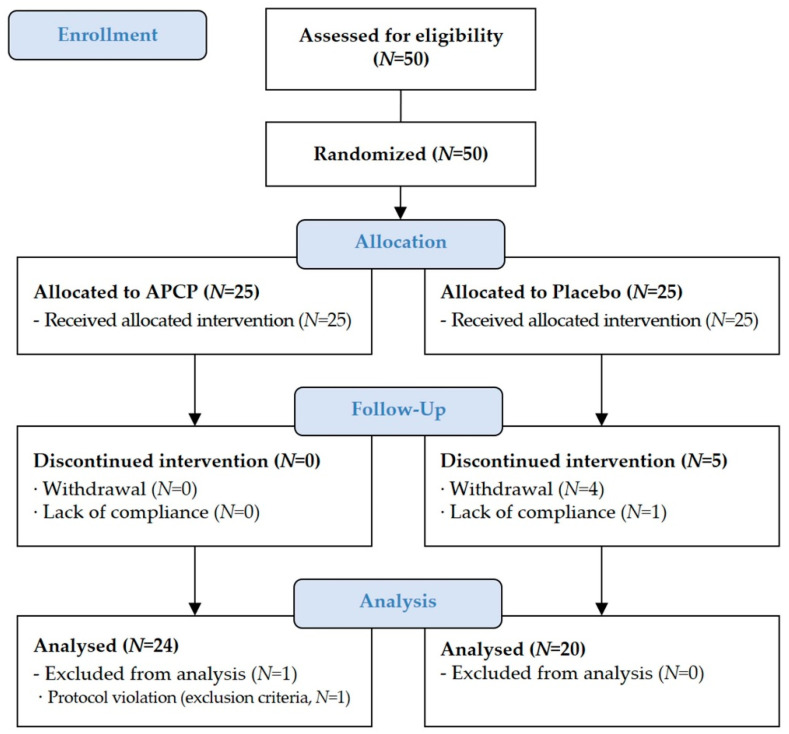
Study flowchart.

The study participants (*N* = 50) were randomized at baseline and allocated to the APCP group (*N* = 25; 1000 mg APCP) or placebo group (*N* = 25). In total, 44 participants completed the trial (Figure 1). Of the six participants who were excluded from the final analysis, one participant was excluded owing to abnormal fasting blood sugar level at the end visit, and five participants dropped out. There were no differences between the APCP and placebo groups in terms of sex, age, body mass index (BMI), and parameter-related skin condition (Table 1). During the study period, no side effects or adverse events were observed.

### 3.2. Skin Hydration

#### 3.2.1. Skin Moisture

**Figure 2:**
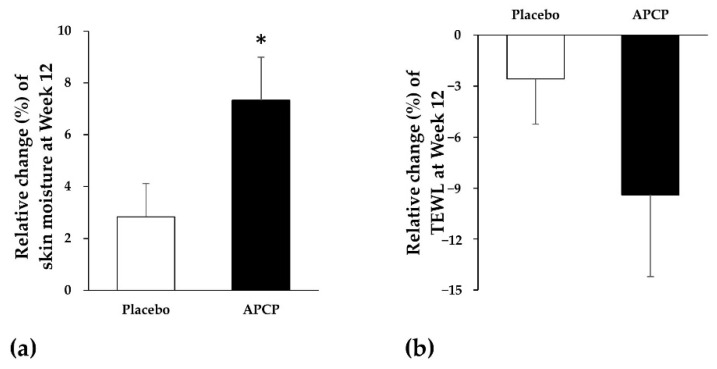
Skin hydration in the forearm. (**a**) Skin moisture and (**b**) transepidermal water loss (TEWL). Relative mean change (%) in the skin moisture and TEWL from baseline after 12 weeks of placebo or APCP ingestion. Data are presented as the mean ± SEM. * *p* < 0.05, as determined using the Mann–Whitney *U* test.

At baseline, the skin moisture in the forearm was 39.32% ± 5.41% and 39.82% ± 6.67% in the APCP and placebo groups, respectively, with no significant difference between the groups. After the 12-week supplementation, the skin moisture in the APCP group was significantly increased by 2.71% ± 3.19% (change over baseline, *p* = 0.002), whereas that in the placebo group exhibited no significant change. Thus, the APCP group (7.33%) showed a significant increase in the relative mean change (%) when compared to the placebo group (2.83%) after 12 weeks (*p* = 0.031; Figure 2a).

#### 3.2.2. TEWL

At baseline, the TEWL from the forearm showed no significant difference between the APCP and placebo groups. The mean TEWL value of the APCP group significantly decreased from 8.17 ± 1.71 g/m^2^h at baseline to 7.36 ± 2.15 g/m^2^h after 12 weeks (*p* = 0.023), whereas the placebo group exhibited no significant change in the TEWL. The relative mean change (%) in the TEWL at 12 weeks was lower in the APCP group (−9.40%) than in the placebo group (−2.58%); however, the difference was not significant (Figure 2b).

### 3.3. Skin Texture

#### 3.3.1. Skin Roughness

**Figure 3:**
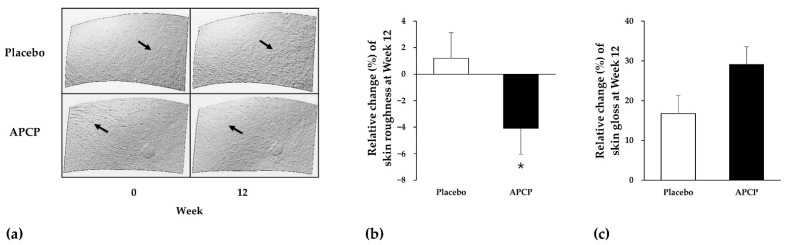
Cheek skin texture (**a**), roughness (**b**), and gloss (**c**). Relative changes in skin roughness and gloss from baseline after 12 weeks of placebo or APCP ingestion. PRIMOS CR Small Field images of the cheek (**a**) of representative participants in the placebo group (upper panels) and APCP group (lower panels) at baseline and after 12 weeks. Data are presented as the mean ± SEM. Skin roughness (**b**) was analyzed using the Mann–Whitney *U* test (* *p* < 0.05), whereas skin gloss (**c**) was analyzed using the independent *t*-test.

In the APCP group, cheek skin roughness decreased from 15.84 ± 2.57 at baseline to 15.18 ± 2.86 at week 12, whereas in the placebo group, it did not significantly change (14.84 ± 2.74 at baseline vs. 14.96 ± 2.69 at week 12). The relative mean change (%) in skin roughness differed significantly between the APCP and placebo groups (−4.09% and 1.21%) at week 12 (*p* = 0.036; Figure 3a,b).

#### 3.3.2. Skin Gloss

At baseline, cheek skin gloss exhibited no significant difference between the groups (65.05 ± 5.86 for the placebo group and 64.31 ± 7.74 for the APCP group). After the intervention period, skin gloss was improved by 16.76% in the placebo group (75.68 ± 13.49, *p* < 0.01) and by 29.13% in the APCP group (82.15 ± 11.58, *p* < 0.001), which was significantly higher (*p* = 0.062; Figure 3c).

#### 3.3.3. Flexibility of the SC

**Figure 4:**
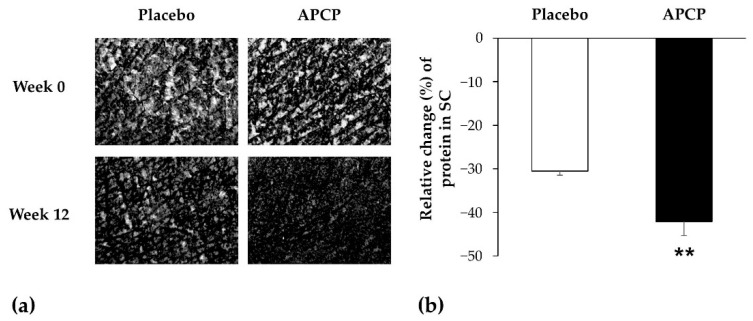
Flexibility of the SC of the forearm. (**a**) Images used for the evaluation of scales on the skin, captured using a Visioscan VC98 instrument. (**b**) Relative changes in the amount of proteins in the SC sampled using the D-squame^®^ tape after 12 weeks. Data are presented as the mean ± SEM. ** *p* < 0.01, as determined using the independent *t*-test.

Changes in the condition of scales on the skin are as shown in Figure 4a. The desquamation index in the APCP group exhibited a decrease from baseline, but no significant difference was noted compared to that of the placebo group (data not shown). Proteins in the SC were quantified to further evaluate skin scaling. The amount of protein in the SC decreased from baseline in the APCP group and showed a significant difference from that in the placebo group at week 12 (*p* = 0.002; Figure 4b).

### 3.4. NMF

#### 3.4.1. AAs

**Figure 5:**
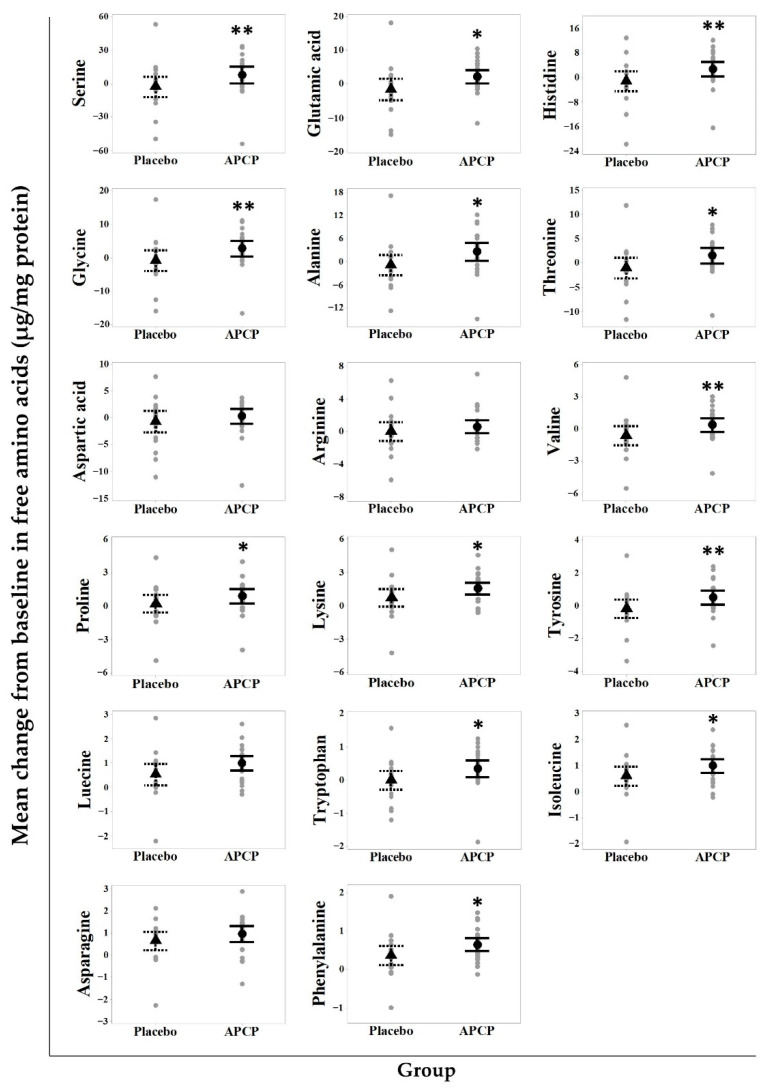
Amino acids (AAs) in the SC. Scatterplot of the changes in AA contents in the SC in the APCP and placebo groups after supplementation. Each gray dot represents a participant. The median is indicated by ▲ (placebo group) or ● (APCP group), and whiskers show the 95% confidence interval. * *p* < 0.05 and ** *p* < 0.01 vs. the placebo group, as determined using the independent *t*-test or Mann–Whitney *U* test.

In total, seventeen AAs were analyzed in SC samples from the forearm. At baseline, the total AA content was 145.25 ± 55.43 μg/mg protein in the APCP group and 135.29 ± 62.16 μg/mg protein in the placebo group, with no statistical difference between the groups (*p* = 0.828). Serine was present at the highest concentration (30.45–31.57%). Glycine, histidine, glutamic acid, and alanine were major constituent AAs, accounting for approximately 10%. After 12 weeks of supplementation, the contents of 13 AA were significantly increased in the APCP group when compared to those in the placebo group (Figure 5). In particular, the mean changes in glycine and proline (two major components of APCP) after APCP ingestion were significantly different from those in the placebo group (*p* = 0.005 and *p* = 0.034).

#### 3.4.2. AADs

The main components of NMF, namely PCA, *trans*-UCA, *cis*-UCA, and citrulline, were analyzed to determine the changes in AADs in the forearm SC after supplementation (Table 2). At baseline, no significant differences in the AAD contents were observed between the APCP and placebo groups. After APCP ingestion, the contents of the AADs in the SC increased, and the PCA, *trans*-UCA, and citrulline contents were significantly different from those in the placebo group, whereas the content of *cis*-UCA was not significantly different. The total amount of these four AADs was significantly higher in the APCP group than in the placebo group.

#### 3.4.3. NMF

**Figure 6:**
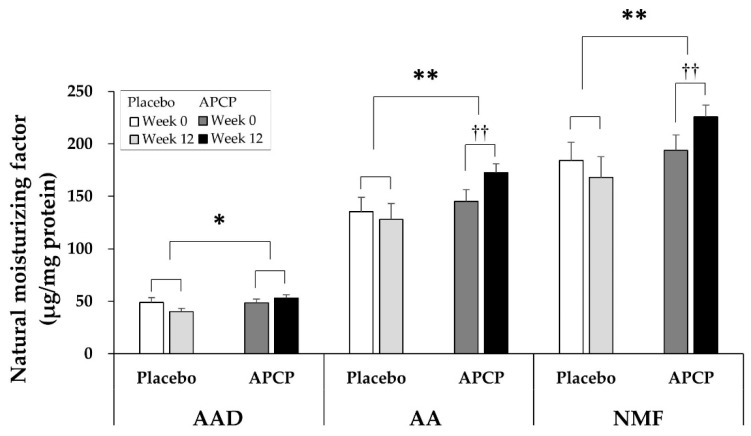
Natural moisturizing factor (NMF) in the SC. The amounts of amino acid derivatives (AADs), AAs, and NMF were normalized to the total amount of protein extracted from the tape-stripped SC samples of the forearm. Data are presented as the mean ± SEM (μg/mg protein). * *p* < 0.05, ** *p* < 0.01 vs. the placebo group, as determined using the independent *t*-test or Mann–Whitney *U* test; †† *p* < 0.01 vs. baseline within each group, as determined using the paired *t*-test or Wilcoxon signed-rank test.

NMF in the SC was calculated as the sum of the amounts of the indicated seventeen AAs and four AADs. The amount of AADs at baseline was 48.85 ± 20.04 μg/mg protein in the placebo group and 48.62 ± 17.54 μg/mg protein in the APCP group, with no significant difference between the groups (*p* = 0.972). After 12 weeks of supplementation, the amount of AADs was 4.68 ± 17.78 μg/mg protein higher in the APCP group than in the placebo group (*p* = 0.016). The total amount of the seventeen AAs exhibited a significant increase from 145.25 ± 55.43 μg/mg protein at baseline to 172.55 ± 41.22 μg/mg protein after 12 weeks of supplementation in the APCP group (*p* = 0.002), which was an increase of 27.30 ± 53.56 μg/mg protein. In the placebo group, the total amount of the seventeen AAs decreased by 7.27 ± 63.75 μg/mg protein from baseline at week 12 and showed a significant difference from that in the APCP group (*p* = 0.007). Consistent herewith, the total amount of NMF in the APCP group significantly increased from 193.87 ± 72.26 at baseline to 225.85 ± 55.24 μg/mg protein after 12 weeks (*p* = 0.007), and the amount of NMF after 12 weeks was significantly greater than that in the placebo group (*p* = 0.006; Figure 6).

### 3.5. Ceramides

**Figure 7:**
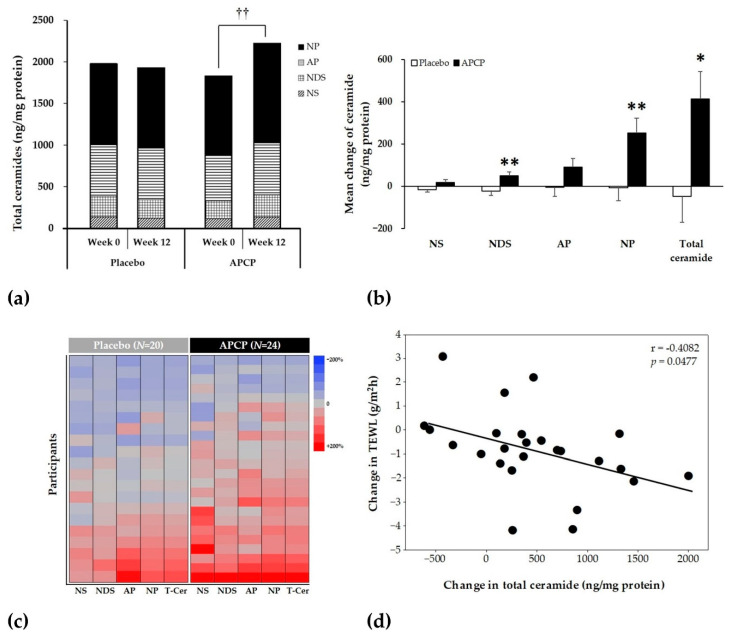
Ceramides in the SC. (**a**) Mean amounts of four ceramide subclasses in the SC. The stacked bar shows the total amount of ceramide at each time point. (**b**) Changes in ceramide contents in the SC from baseline after 12 weeks in the placebo and APCP groups. (**c**) Heatmap showing relative changes in skin ceramide contents in the placebo (left) and APCP (right) groups. The relative change in the level of each ceramide subclass is plotted for each participant. (**d**) Correlation between changes in the total ceramide content and TEWL over 12 weeks in individual participants in the APCP group (*n* = 24). The line indicates the optimal linear fit through all data points. The r value is the Pearson correlation coefficient. Data are presented as the mean ± SEM (ng/mg protein). †† *p* < 0.01 vs. baseline within APCP group, as determined using the paired *t*-test. * *p* < 0.05, ** *p* < 0.01 vs. the placebo group, as determined using the independent *t*-test. NS, a non-hydroxy FA conjugated to sphingosine; NDS, a non-hydroxy FA conjugated to dihydrosphingosine base; NP, a non-hydroxy FA conjugated to phytosphingosine; AP, α-hydroxy FA conjugated to phytosphingosine; T-cer, total ceramides, i.e., the sum of the contents of NS, NDS, NP, AP.

To evaluate the changes in ceramide content in the SC after supplementation, the contents of NP, NS, NDS, and AP were determined. At baseline, the total ceramide content (calculated as sum of the contents of the four ceramide subclasses) was 1842.47 ± 463.86 ng/mg protein in the APCP group and 1993.11 ± 761.89 ng/mg protein in the placebo group, with no significant difference between the groups (Figure 7a). The mean total ceramide content in the APCP group significantly increased from 1842.47 ± 463.86 ng/mg protein at baseline to 2256.58 ± 651.23 ng/mg protein after 12 weeks (*p* = 0.003; Figure 7a), whereas the placebo group exhibited no significant change. The changes in the contents of the four ceramide subclasses after 12 weeks of supplementation are shown in Figure 7b; the NDS, NP, and total ceramide contents were significantly higher in the APCP group than in the placebo group. The percent changes in the four ceramide subclass and total ceramide contents during the intervention period for each participant are presented as a heatmap in Figure 7c; in the placebo group, the numbers of participants with increased or decreased values were similar, whereas in the APCP group, the number of participants with increased values was higher than the number of participants with decreased values. The improvement in total ceramide content was 79.17% in the APCP group, which was significantly higher than that in the placebo group (45%; *p* = 0.018). Moreover, changes in the total ceramide content and TEWL were negatively correlated (r = −0.408, *p* = 0.048) in the APCP group (Figure 7d).

## 4. Discussion

In this study, we demonstrated that the ingestion of APCP can promote skin barrier function and skin hydration. Furthermore, we showed that the improved skin moisturization and reduced SC scaling in the APCP group may have been related with an increase in NMF. The total contents of AAs and AADs of the SC were significantly increased in the APCP group compared with those in the placebo group. In particular, the levels of Gly and Pro, the major AAs of fish collagen [44], were significantly increased, which may be attributable to the intake of the *N. virgatus*-derived APCP. As mentioned in the Introduction, collagen peptide intake induces the production of filaggrin [37]. Filaggrin is completely decomposed into free AAs, some of which are further derivatized, in the uppermost layer of the SC [40]. Glutamine is nonenzymatically transformed into PCA, which has high hygroscopicity [38,45]. The increase in PCA in the APCP group may have contributed to the maintenance of moisture in the SC. Histidine is converted into UCA by histidase [38]. The content of *trans*-UCA, which protects the skin from the harmful effects of solar UV radiation, was increased in the APCP group as compared to that in the placebo group, whereas the content of *cis*-UCA was not. This is because *trans*-UCA is a derivative of histidine, whereas *cis*-UCA is generated by photoisomerization of *trans*-UCA after UV radiation exposure [46]. Arginine is converted to citrulline through enzymatic deimination by peptidylarginine deiminases [47]. Citrulline levels were found to be significantly increased in the APCP group when compared with those in the placebo group. The deimination of arginine by peptidylarginine deiminases promotes the breakdown of filaggrin into free AAs, which are thought to be important for water retention in corneocytes [4,38]. A recent study by Miyanaga et al. [48] demonstrated that the improvement in skin moisture content after oral ingestion of collagen peptides could be attributed to increases in NMF component levels (i.e., increases in the levels of total AAs, PCA, and UCA) in the SC. These results suggest that APCP intake increases and maintains skin moisture by increasing the levels of NMF components, such as AAs and AADs, in the SC.

The major role of the SC is to form a skin barrier. The effectiveness of the skin barrier is affected by the concentration of intercellular lipids in the SC, the most important of which is ceramide [7]. These lipids surround the keratinocytes in the extracellular space of the SC and form a continuous phase that retains moisture. Several studies have shown that with aging and in skin diseases, such as AD and psoriasis, ceramide levels in the SC decrease, leading to skin barrier damage and dry skin [11,49,50,51]. Among the major ceramides found in the SC, the contents of NP, NDS, a non-hydroxy FA conjugated to 6-hydroxy sphingosine, α-hydroxy FA conjugated to 6-hydroxy sphingosine, and total ceramide are significantly negatively correlated with TEWL [52]. In particular, the levels of NP and NDS are negatively correlated, whereas that of NS containing short acyl chains is positively correlated with TEWL values [53,54,55]. In a mouse photoaging model, skin ceramide levels were reduced by UV irradiation, whereas fish collagen administration rescued the decrease in ceramide to a level similar to that in the normal control group [35]. In human keratinocytes treated with APCP, the level of the ceramide synthetase serine palmitoyltransferase increased [56]. Based on the results of these studies, we investigated the effect of APCP intake on the ceramide level in the SC by measuring the changes in the amounts of NP, NDS, AP, NS, and total ceramide in the SC of the study participants. The amount of each ceramide subclass increased with APCP ingestion, but the levels of only the NP and NDS were increased significantly when compared with those in the placebo group. The amount of total ceramide in the APCP group was significantly increased when compared with that in the placebo group. Consistent with the results of previous studies, the ceramide content and TEWL exhibited a significant negative correlation in the APCP group. This study demonstrated that the permeability barrier function of the SC can be improved through an increase in the ceramide content in the SC, which can be achieved by ingesting APCP. A decrease in moisture in the SC leads to loss of flexibility of the SC, as indicated by increased roughness due to scales on the skin surface and decreased skin gloss [18]. APCP intake improved the skin texture by decreasing the skin roughness, the number of scales on the skin, and SC protein content, and increasing skin gloss. This clinical improvement indicates that APCP intake has a positive effect on the flexibility of the SC.

This study demonstrated that oral intake of APCP decreased TEWL and increased skin moisture content and increases in the ceramide and NMF contents. The clinical findings of increased skin moisture and reduced skin roughness and TEWL may explain the improvements in SC flexibility and the skin barrier function. One major limitation of this study was that we only analyzed ceramides, not cholesterol and FAs, among the SC lipids. Different analytical methods had to be used for NMF and ceramides, and for the safety of the participants, the SC was sampled from only one part of the forearm. Therefore, ceramides, which are known to be the most important for skin barrier, were analyzed first. In future, we plan to investigate the improvement in skin barrier function by APCP and their mechanisms of action by analyzing the levels of skin barrier-related lipids. Despite this limitation, to the best of our knowledge, the present study is the first to demonstrate an increase in the skin moisture content and improvement of the skin barrier function through the simultaneous analysis of NMF and ceramides in the SC after oral intake of APCP in adult men and women.

## 5. Conclusions

In conclusion, APCP intake in dry skin helps to increase skin moisture and improve the skin barrier function by increasing NMF and ceramide contents in the SC.

## Figures and Tables

**Table 1:** Baseline characteristics of the study participants.

	Placebo (*N* = 25)	APCP (*N* = 25)	*p*
Sex–Female (%)	14 (56.00%)	14 (56.00%)	1.000 ^2^
Male (%)	11 (44.00%)	11 (44.00%)	
Age (years)	47.80 ± 5.82 ^1^	48.40 ± 5.41	0.708 ^3^
BMI ^4^ (kg/m^2^)	24.08 ± 2.99	24.09 ± 4.07	0.994 ^3^
Skin moisture (%)	39.45 ± 6.64	39.49 ± 5.37	0.981 ^3^
TEWL ^4^ (g/m^2^h)	8.59 ± 1.69	8.19 ± 1.68	0.408 ^3^
Skin texture (Ra)	14.77 ± 2.62	15.95 ± 2.59	0.114 ^3^
Skin gloss (SGU)	64.01 ± 7.47	64.39 ± 7.59	0.857 ^3^

^1^ Data are presented as the mean ± SD. ^2^ The *p*-value for the difference in the numbers of women and men between the placebo and APCP groups was calculated using the chi-square test. ^3^ The *p*-value for the difference between the placebo and APCP groups was calculated using the independent *t*-test or Mann–Whitney *U* test. ^4^ BMI, body mass index; TEWL, transepidermal water loss.

**Table 2:** Relative amounts of AADs in the SC.

AAD.	Week	Placebo (*N* = 20)	APCP (*N* = 24)
PCA	0	25.18 ± 11.04	26.41 ± 10.43
	12	20.21 ± 9.44	28.27 ± 8.16
	change	−4.97 ± 9.43	1.86 ± 9.64 *
*trans*-UCA	0	8.78 ± 4.96	9.18 ± 3.87
	12	7.67 ± 4.44	10.69 ± 3.99
	change	−1.11 ± 3.54	1.51 ± 4.43 *
*cis*-UCA	0	0.03 ± 0.05	0.05 ± 0.09
	12	0.11 ± 0.10	0.15 ± 0.18
	change	0.08 ± 0.10	0.10 ± 0.20
Citrulline	0	14.86 ± 6.65	12.99 ± 3.91
	12	12.04 ± 6.90	14.04 ± 4.51
	change	−2.82 ± 6.60	1.05 ± 4.77 *
Sum of AADs	0	48.85 ± 20.04	48.62 ± 17.54
	12	40.04 ± 14.71	53.15 ± 14.71
	change	−8.74 ± 18.50	4.68 ± 17.78 *

* *p* < 0.05 vs. the placebo, as determined using the independent *t*-test or Mann–Whitney *U* test. Data are presented as the mean ± SD (μg/mg protein).

## Data Availability

Data sharing is not applicable to this article due to ethical policy.
